# Sheep recombinant IGF-1 promotes organ-specific growth in fetal sheep

**DOI:** 10.3389/fphys.2022.954948

**Published:** 2022-08-25

**Authors:** J Stremming, A White, A Donthi, DG Batt, B Hetrick, EI Chang, SR Wesolowski, MB Seefeldt, CE McCurdy, PJ Rozance, LD Brown

**Affiliations:** ^1^ Department of Pediatrics, Perinatal Research Facility, University of Colorado Anschutz Medical Campus, Aurora, CO, United States; ^2^ Gates Biomanufacturing Facility, University of Colorado Anschutz Medical Campus, Aurora, CO, United States; ^3^ Department of Human Physiology, University of Oregon, Eugene, OR, United States

**Keywords:** fetal growth, skeletal muscle, myoblast, IGF-1 signaling, IGF binding protein

## Abstract

IGF-1 is a critical fetal growth-promoting hormone. Experimental infusion of an IGF-1 analog, human recombinant LR3 IGF-1, into late gestation fetal sheep increased fetal organ growth and skeletal muscle myoblast proliferation. However, LR3 IGF-1 has a low affinity for IGF binding proteins (IGFBP), thus reducing physiologic regulation of IGF-1 bioavailability. The peptide sequences for LR3 IGF-1 and sheep IGF-1 also differ. To overcome these limitations with LR3 IGF-1, we developed an ovine (sheep) specific recombinant IGF-1 (oIGF-1) and tested its effect on growth in fetal sheep. First, we measured *in vitro* myoblast proliferation in response to oIGF-1. Second, we examined anabolic signaling pathways from serial skeletal muscle biopsies in fetal sheep that received oIGF-1 or saline infusion for 2 hours. Finally, we measured the effect of fetal oIGF-1 infusion versus saline infusion (SAL) for 1 week on fetal body and organ growth, *in vivo* myoblast proliferation, skeletal muscle fractional protein synthetic rate, *IGFBP* expression in skeletal muscle and liver, and IGF-1 signaling pathways in skeletal muscle. Using this approach, we showed that oIGF-1 stimulated myoblast proliferation *in vitro*. When infused for 1 week, oIGF-1 increased organ growth of the heart, kidney, spleen, and adrenal glands and stimulated skeletal myoblast proliferation compared to SAL without increasing muscle fractional synthetic rate or hindlimb muscle mass. Hepatic and muscular gene expression of *IGFBPs one to three* was similar between oIGF-1 and SAL. We conclude that oIGF-1 promotes tissue and organ-specific growth in the normal sheep fetus.

## Introduction

Insulin-like growth factor-1 (IGF-1) is a critical growth hormone that primarily promotes growth in the fetus. Umbilical cord blood concentrations of IGF-1 at birth have a strong positive correlation with birth weight in humans (Gluckman et al., 1983; Lassarre et al., 1991; Ong et al., 2000; Christou et al., 2001; Patel et al., 2018). Pregnancies that are complicated by fetal macrosomia or intrauterine growth restriction (IUGR) have either high or low fetal IGF-1 concentrations, respectively (Roth et al., 1996; Ostlund et al., 1997). Humans and rodents with IGF-1 or IGF-1 receptor gene mutations demonstrate significant growth restriction (Liu et al., 1993; Woods et al., 1996; Abuzzahab et al., 2003; Fowden, 2003).

IGF-1 is synthesized by the fetus and is present in most fetal tissues in humans, rodents, and cows (Lund et al., 1986; Han et al., 1988; Chard, 1994; Ghanipoor-Samami et al., 2018). In the circulation, most IGF-1 is bound by six major IGF binding proteins (IGFBP) (Bach, 2018). IGFBPs regulate the activity of IGF-1 and IGF-2. IGFBPs typically attenuate IGF activity, but some IGFBPs potentiate IGF activity. IGFBPs may also extend the half-life of circulating IGF-1 (Guler et al., 1989; Allard and Duan, 2018; Bach, 2018). In adults, IGF-1 is primarily bound to IGFBP-3 and an acid labile glycoprotein subunit, which extends the half-life of IGF-1 from minutes to hours (Allard and Duan, 2018; Bach, 2018). At birth, fetal cord blood concentrations of IGFBP-1 and IGFBP-3 correlate negatively and positively, respectively, with birth weight as corrected for gestational age (Ong et al., 2000). In the setting of IUGR, umbilical cord concentrations of IGFBP-3 are low, and concentrations of IGFBP-1 are high; the reverse is true in fetal macrosomia (Giudice et al., 1995; Cianfarani et al., 1998; Wiznitzer et al., 1998). Other IGFBPs, including 2 and four to six, are also present in the fetus and umbilical circulation (Chard, 1994; Giudice et al., 1995; Ghanipoor-Samami et al., 2018).

In mammals, including humans, rodents, and sheep, the mature IGF-1 protein contains 70 amino acids, but there is some variation in the amino acid sequence (Rotwein, 2017). Despite differences in human and animal IGF-1 peptide sequences, recombinant human long arginine 3 IGF-1 (LR3 IGF-1) is commonly used to test the physiological effects of IGF-1 in animal models. LR3 IGF-1 is a larger protein than native human or sheep IGF-1, containing 83 amino acids instead of 70 with the additional 13 amino acids on the N-terminus of the peptide. Additionally, the third amino acid in the native human or sheep sequence, glutamic acid, is replaced with an arginine in LR3 IGF-1, thus substituting a negatively charged amino acid in place of a positively charged amino acid. This analog of IGF-1 has a strong affinity for IGF-1 receptors but a low affinity for IGFBPs. In cultured rat myoblasts, LR3 IGF-1 is highly potent in stimulating protein synthesis and attenuating protein breakdown (Francis et al., 1992) compared to recombinant IGF-1. However, in adult rats, LR3 IGF-1 is cleared from the plasma more rapidly than recombinant human IGF-1 (Bastian et al., 1993). Infusion of LR3 IGF-1 into the sheep fetus does not consistently increase fetal body weight or length. However, fetal LR3 IGF-1 infusion for seven to 10 days does consistently increase the weight of specific organs, including the heart, spleen, and adrenal glands (Lok et al., 1996; Sundgren et al., 2003; Stremming et al., 2021). We also have demonstrated that LR3 IGF-1 infusion for 1 week increases fetal skeletal myoblast proliferation but not muscle weight (Stremming et al., 2021).

Because of these differences between human recombinant LR3 IGF-1 and sheep IGF-1 and the utility of using pregnant sheep to study the critical roles of IGF-1 in the regulation of fetal growth, we developed an ovine (sheep) specific IGF-1 (oIGF-1). Native IGF-1, such as oIGF-1 in the sheep fetus, should be subject to regulation by IGFBPs, thus mimicking the physiologic regulation of IGF-1. We hypothesized that oIGF-1 would promote organ-specific growth effects in fetal sheep. To test this hypothesis, we utilized a three-pronged approach. First, myoblasts from late gestation fetal sheep were exposed to oIGF-1 to measure myoblast proliferation *in vitro*. Second, fetal sheep were infused with oIGF-1 for 2 hours, during which serial muscle biopsies were obtained from biceps femoris (BF) muscle to measure molecular signaling responses to oIGF-1. Finally, fetal sheep were infused with oIGF-1 for 1 week to measure the effects of oIGF-1 on fetal body growth, organ growth, skeletal myoblast proliferation, and hypertrophic muscle growth *in vivo*.

## Materials and methods

### Ethical approval

All study protocols were approved the by Institutional Animal Care and Use Committee at the University of Colorado Anschutz Medical Campus (Protocol numbers 77617(10)1E, 00470, and 00334) and are in compliance with guidelines from AAALAC International. Experiments were performed at the Perinatal Research Facility on the Anschutz Medical Campus. Experiments are in compliance with the ARRIVE guidelines (Percie du Sert et al., 2020).

### Expression and purification of recombinant oIGF-1

**FIGURE 1:**
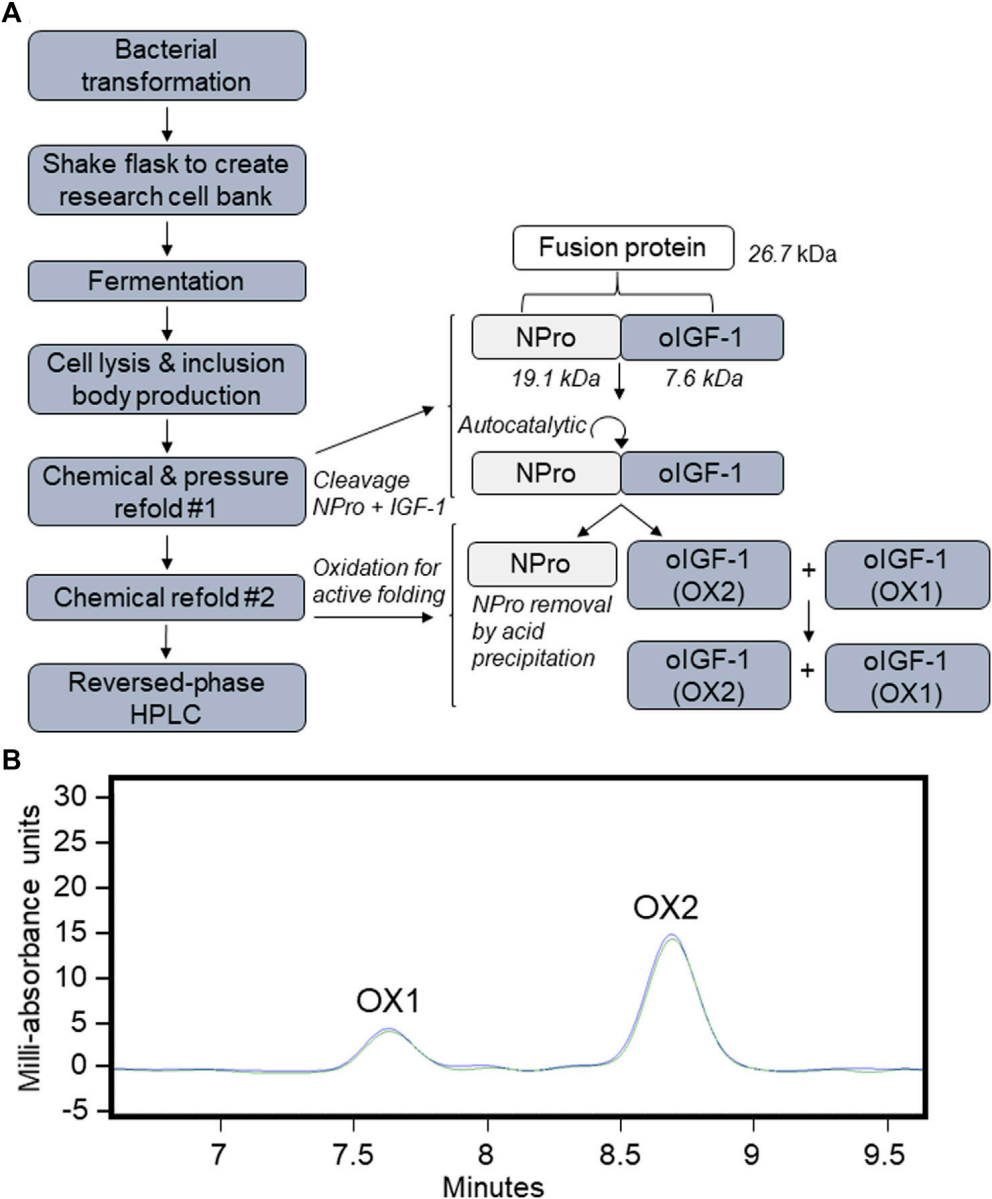
Recombinant ovine IGF-1 (oIGF-1) production. **(A)** Process flow of expression and purification. **(B)** Preparative reversed-phase HPLC chromatography and elution profiles of misfolded oxidation 1 (OX1) and biologically active oxidation 2 (OX2) forms.

The recombinant oIGF-1-N-terminal-protease (NPro) fusion protein coding sequence was cloned into pD451-SR plasmid under T7 promoter and transformed into *E. coli* BL21(DE3). Transformed cells were grown in high density fed-batch fermentation, and recombinant oIGF was expressed as insoluble inclusion bodies. After the harvest, the cells were lysed and washed several times to prepare inclusion bodies. The inclusion bodies were solubilized in denaturing conditions and refolded by chemical methods resulting in active NPro that self-cleaves the oIGF-1 fusion partner. Refolded protein was precipitated in the presence of PEG-8000, resolubilized in denaturing conditions, and chemically refolded in the presence of organic containing buffer. NPro fusion partner was removed by acid precipitation leaving soluble oIGF-1 mixture of misfolded oxidation 1 (OX1) and biologically active oxidation 2 (OX2). The solution was buffer exchanged through tangential flow filtration to remove residual organics to eliminate interference for downstream purification. Further purification by preparative reversed-phase HPLC resulted in separation of OX1 and OX2 forms. The purified OX2 was buffer exchanged into a final formulation buffer at a protein concentration appropriate to downstream applications. An overall process flow and an elution profile of oIGF-1 from reversed-phase HPLC is shown in Figure 1.

### Cell culture to assess myoblast proliferation

We first tested the proliferative capacity of oIGF-1 *in vitro* by exposing primary skeletal myoblasts to oIGF-1. BF skeletal muscle biopsies were obtained from six normally growing late gestation fetal sheep (132 ± 0 days gestation, term gestation is 147 days) and cultured as previously described (Soto et al., 2017) with the following exceptions. Myoblasts were thawed and passaged, and studies were performed at passage 3. Experiments were carried out in eight replicates per animal, which were averaged together. Myoblasts were plated at 5,000 myoblasts per well in a 96-well plate in Skeletal Muscle Cell Growth Medium with SupplementMix (PromoCell, Heidelberg, Germany, Cat. No. C-23060); SupplementMix components used include: fetal bovine serum (FBS), fetuin, epidermal growth factor, and dexamethasone. Gibco™ Antibiotic-Antimycotic 100X was also added (Thermo Fisher Scientific, Waltham, MA, Cat. No. 15240062). Myoblasts were starved for 24 h in Dulbecco’s Modified Eagle’s Medium (DMEM, Thermo Fisher Scientific), then they were treated with one of the following conditions for 72 h: DMEM, DMEM with 10% FBS, DMEM with 1 ng mL^−1^ oIGF-1, or DMEM with 10 ng mL^−1^ oIGF-1. Cellular proliferation was measured using a 3-(4,5-dimethylthiazol-2-yl)-2,5-diphenyl tetrazolium bromide (MTT) assay (EMD Millipore Corp., Billerica, MA; Cat. No. CT02).

### Surgical procedures for intraoperative oIGF-1 infusion

To test the acute effects of oIGF-1 on anabolic signaling pathways, pregnant Columbia-Rambouillet mixed-breed sheep with ewes carrying either a singleton fetus (n = 1) or twin fetuses (n = 3), underwent maternal laparotomy and hysterotomy under general anesthesia for fetal catheter placement at 136 ± 1 day gestation. Anesthesia was induced as previously described (Rozance et al., 2018; Stremming et al., 2021), and the animals were maintained under general anesthesia for the duration of the procedure. A maternal laparotomy and hysterotomy were performed, and a fetal forelimb was exposed for placement of polyvinyl catheters in the brachial artery and vein. The venous catheter was used for infusions, and the arterial catheter was used to obtain blood samples as described below. Prior to infusion start, two baseline blood samples were obtained. oIGF-1 was infused into the singleton animal in order to develop a dose response curve. oIGF-1 in 0.5% bovine serum albumin (BSA) was first infused at 22 μg h^−1^. Blood samples were obtained at 15, 30, 60, and 115 min of the infusion. At 120 min, the infusion rate was increased to 220 μg h^−1^, and blood samples were obtained at 135, 150, 180, and 235 min. At 240 min, the infusion rate was increased to 440 μg h^−1^, and a final blood sample was obtained at 255 min. For the twin fetuses, arterial and venous catheters were placed in each twin as described above. The fetal hindlimbs were then exposed, and a muscle biopsy was obtained from the BF muscle. The hindlimbs were then covered with drapes soaked in warm saline and a heating blanket in between BF biopsies. One twin was randomly assigned to receive an infusion of oIGF-1 at either 22 μg h^−1^ (n = 1) or 220 μg h^−1^ (n = 2), and the other twin received a volume matched infusion of saline in 0.5% BSA (n = 3). Muscle biopsies and blood samples were obtained at baseline prior to infusion start and at 5, 15, 30, 60, 90, and 120 min. At the conclusion of the surgical procedure, while still under anesthesia, the ewe and each fetus were given a fatal dose of intravenous pentobarbital (Fatal Plus, Vortech Pharmaceuticals, Dearborn, MI). Tissue samples from this cohort of animals will be described as “acute” samples.

### Surgical procedures for 1-week oIGF-1 infusion

To test the chronic effects of oIGF-1 on fetal growth and anabolic signaling pathways, pregnant Columbia-Rambouillet mixed-breed sheep (n = 6) carrying twin fetuses underwent maternal laparotomy and hysterotomy under general anesthesia for fetal catheter placement as previously described (Rozance et al., 2018; Stremming et al., 2021). Polyvinyl catheters were placed in each twin in the bilateral fetal pedal arteries with the tip positioned in the external iliac artery and bilateral fetal saphenous veins with the tip positioned in the common femoral vein, and in the maternal femoral artery and maternal femoral vein. A 0.5 cm^3^ biopsy was obtained from the BF muscle of each fetus and frozen at −70 °C in order to measure baseline isotopic enrichment of phenylalanine prior to tracer infusion.

Animals were allowed to recover for at least 6 days prior to infusion start. Within each twin pair, one twin was randomly assigned to receive an infusion of oIGF-1 in 0.5% BSA in saline (25 μg kg^−1^•hr^−1^ based on an estimated fetal weight of 3.5 kg) and the other twin received a volume matched infusion of 0.5% BSA in saline (SAL). We have previously shown that LR3 IGF-1 has growth promoting effects in fetal sheep at a dose of 6.6 μg kg^−1^•hr^−1^ (Stremming et al., 2021). However, because oIGF-1 will bind to IGFBPs, we selected a higher dose of 25 μg kg^−1^•hr^−1^. This is a similar dose to that of recombinant human IGF-1 that was shown to promote growth in fetal sheep (Lok et al., 1996). Fetal arterial blood samples were obtained at baseline prior to infusion start and then daily for measurements of fetal blood gas, glucose, lactate, insulin, and IGF-1 concentrations. Fetal blood concentrations of amino acids, cortisol, and norepinephrine were measured at baseline and again on the final day of the infusion. Twenty-4 hours prior to necropsy, the fetus was given a 10 mg kg^−1^ bolus of 5-ethynyl-2′-deoxyuridine (EdU; Thermo Fisher Scientific, Cat. No. A10044) intravenously to measure myoblast proliferation. At this time, fetal infusions of two isotopomers of phenylalanine, Phe ring ^2,3,3,2^H_8_ (m+8) and Phe ring ^2^H_5_ (m+5) were started 6 hours apart, both at a rate of 2 ml h^−1^, to measure fractional muscle protein synthetic rates as previously described (Rozance et al., 2018).

After conclusion of the metabolic study, the ewe received diazepam (0.2 mg kg^−1^) and ketamine (20 mg kg^−1^) intravenously. The fetus was delivered via maternal laparotomy and hysterotomy. A BF muscle biopsy was obtained from the fetal hindlimb; a portion of the biopsy was immediately frozen in liquid nitrogen, and a portion of the biopsy was placed in Ham’s F-12 Nutrient Mix media (Thermo Fisher Scientific, Cat. No. 11765047) for single cell suspension as described below. A liver biopsy was obtained and immediately frozen. A lethal dose of pentobarbital sodium (Vortech Pharmaceuticals, Dearborn, MI) was administered intravenously to the ewe and the fetus. Fetal body and organ weights were obtained. Fetal hindlimb muscles, including BF, gastrocnemius, tibialis anterior, and flexor digitorum superficialis, were quickly dissected from the hindlimb, weighed, and snap frozen in liquid nitrogen. Tissue samples from this cohort of animals will be described as “chronic” samples.

### Fetal blood measurements

Fetal blood was analyzed for pH, P_a_CO_2_, P_a_O_2_, oxygen saturation, oxygen content, and hematocrit using a blood gas analyzer (ABL 825, Radiometer, Copenhagen, Denmark). Fetal plasma was used to measure glucose and lactate concentrations (YSI 2900, YSI Inc., Yellow Springs, OH). Plasma insulin, IGF-1, and cortisol concentrations were measured by ELISA as previously described (White et al., 2021). Plasma amino acid and norepinephrine concentrations were measured using HPLC as previously described (Limesand et al., 2006). Muscle enrichment of the two isotopomers of phenylalanine was measured in order to calculate fractional synthetic rate (FSR) as previously described (Rozance et al., 2018).

### Single cell suspensions for flow cytometry

BF muscle from the chronically infused animals was rinsed twice in Hanks’ Balanced Salt solution (HBSS, Thermo Fisher Scientific, Cat. No. 14175095). Tissue was placed in a sterile tissue culture plate with 5 ml of 2% collagenase in HBSS. Non-muscle tissue was carefully dissected from the sample, and then the sample was minced into small pieces. An additional 5 ml of HBSS was added for a final collagenase concentration of 1%. The muscle was digested in 1% collagenase in HBSS for 30 min at 37°C with constant rotation. An equivalent volume of 0.5% BSA in HBSS was added in order to neutralize the collagenase, and then the sample was strained through a 70 μm cell strainer. After straining, the sample was centrifuged at 300 x g for 10 min at room temperature. The cell pellet was collected and resuspended in 10 ml of 1X phosphate buffered saline (PBS) and washed with additional 1X PBS up to 50 ml. This was centrifuged again as above. The cell pellet was collected and resuspended in 10 ml of 1X PBS, and the cells were counted. Additional 1X PBS was added to a final cell concentration of 1 × 10^6^ cells•mL^−1^. Yellow Live/Dead stain (Thermo Fisher Scientific, Cat. No. L34968) was added at a ratio of 1 µL for every 10^6^ cells and incubated in the dark for 30 min at room temperature. The sample was then centrifuged at 300 x g for 10 min at room temperature. The cell pellet was then washed with 1X PBS. Cells were fixed in 2% paraformaldehyde in 1X PBS at a concentration of 1 × 10^6^ cells•mL^−1^ for 15 min on ice. Cells were centrifuged at 300 x g for 10 min at room temperature. The cell pellet was collected and washed with 1X PBS; the sample was then centrifuged again as above. The cell pellet was then resuspended in 1X PBS at a concentration of 1 × 10^6^ cells•mL^−1^.

### Flow cytometry

Flow cytometry was completed at the University of Oregon in Eugene, OR. Paraformaldehyde fixed myoblasts were washed into ice cold 70% ethanol and stored overnight at 4°C. Myoblasts were then washed with 1X PBS, and incorporated EdU was labeled using Click-iT Plus EdU Alexa Fluor 647 Flow Cytometry Assay Kit (Thermo Fisher Scientific, Cat. No. C10634) according to the manufacturer’s instructions. Cells were then washed in incubation buffer (1% BSA in 1X PBS) and stained for myogenic lineage using 1 μg of CD56 primary antibody conjugated to PE/Cyanine5 (BioLegend, San Diego, CA, United States, Cat. No. 304607; RRID: AB_314449) in 0.1 ml incubation buffer for 30 min at 25°C. Myoblasts were then washed into 0.5 ml incubation buffer with 3 µM DAPI. All wash steps were carried out by centrifugation at 500 *g* for 5 min at 25°C followed by decanting the supernatant. Data was acquired using a Gallios 3-laser flow cytometer and Kaluza software version 2.1 (Beckman Coulter, Indianapolis, IN, United States). Compensation was performed using singly stained cell preparations. Gates were determined by comparing unstained and singly stained samples. Percent EdU positive cells were analyzed within the Yellow Live/Dead negative, DAPI positive, and CD56 positive subset.

### Western blot

BF muscle from both the acute (intraoperative) and chronic (1 week) oIGF-1 infusion groups was homogenized in lysis buffer (Cell Signaling, Danvers, MA) with protease and phosphatase inhibitors (Sigma-Aldrich, St. Louis, MO). Protein concentration of lysates was measured using Pierce BCA Protein Assay (Thermo Fisher Scientific). 25 μg of protein was combined with dithiothreitol and loaded onto a precast 4–12% bis-tris gel (Thermo Fisher Scientific). Electrophoresis was performed, and protein was transferred to nitrocellulose membranes (GVS, Sanford, ME). Membranes with samples from animals that received a 1-week infusion of oIGF-1 were stained for protein using Revert 700 Total Protein Stain (LI-COR Biosciences, Lincoln, NE), which was used to account for any differences in loading and transfer; membranes with tissue samples from animals that received an acute oIGF-1 infusion were stained for beta actin (MP Biomedicals, Irvine, CA, Cat. No. 691002, RRID: AB_2335304). Membranes were blocked in 5% non-fat milk in tris-buffered saline with 0.1% Tween (TBST) overnight at 4°C and subsequently incubated with primary antibodies in 5% BSA in 1X TBST overnight at 4°C. Primary antibodies included: total protein kinase B (Akt) (Cell Signaling Cat. No. 9272, RRID: AB_329827), phosphorylated Akt (Ser 473) (Cell Signaling Cat. No. 9271, RRID: AB_329825), total extracellular signal-regulated kinase (Erk 1/2) (Cell Signaling Cat. No. 9102, RRID: AB_330744), and phosphorylated Erk 1/2 (Thr202/Tyr204) (Cell Signaling, Cat. No. 9101, RRID_AB:331646). Primary antibodies were diluted to 1:1,000. Bands were visualized using near-infrared fluorescence exposed on Odyssey FC-0788 (LI-COR Biosciences) using the appropriate goat anti-mouse (LI-COR Cat. No. 925-68070, RRID: AB_2651128) or goat anti-rabbit (LI-COR Cat. No. 926-32211, RRID: AB_621843) secondary antibody (1:5,000) in 5% non-fat milk in 1X TBST.

### Real-time qPCR

BF muscle and liver biopsies obtained from animals that received a 1-week infusion of either oIGF-1 or SAL were used for real-time qPCR. RNA was extracted and real-time PCR was performed as previously described (Thorn et al., 2013; Brown et al., 2015). PCR primers for IGF related genes were optimized and are listed in Table 1. Quantitative real-time qPCR was performed using the LightCycler 480 (Roche Life Science, Indianapolis, IN). Expression of the target genes was normalized to the geometric mean of 3 reference genes in BF (hydroxymethylbilan synthase, ribosomal protein L37a, and ribosomal protein S15) and 3 reference genes in the liver (beta-2-microglobulin, hydroxymethylbilan synthase, and ribosomal protein S15). There were no differences in expression of any of the reference genes between SAL and oIGF-1.

**TABLE 1:** Real-time qPCR primers.

Gene	Forward	Reverse	Accession number
Insulin-like growth factor 1 (*IGF-1*)	GAG​ACC​CTC​TGC​GGG​GCT​GA	CTG​CTC​GAG​CCG​TAC​CCC​GT	NM_001009774.3
Insulin-like growth factor 2 (*IGF-2*)	TGT​GGG​GAC​CGC​GGC​TTC​TA	CAG​GGC​CAG​GTC​GCA​GCT​TC	XM_027959253.1
Insulin-like growth factor 1 receptor (*IGF-1R*)	TGT​CCT​GAC​ATG​CTG​TTT​GAG​CTG	CCA​GGA​ACG​AGG​GCC​GCA​TC	AF025303
Insulin-like growth factor 2 receptor (*IGF-2R*)	ACC​AGT​TAC​GCC​TGC​CCG​GA	TCG​GGA​CCG​CCC​TCG​GAT​TT	AF353513
IGF binding protein 1 (*IGFBP-1*)	GCC​AGG​GAG​CAG​CAG​AAG​GC	GAG​CCC​AGG​CTC​TCC​GTC​CA	NM_001145177.1
IGF binding protein 2 (*IGFBP-2*)	ACC​TTG​GCC​TGG​AGG​AGC​CC	TCC​AGG​GGA​CCC​CGC​TCA​TC	NM_001009436.1
IGF binding protein 3 (*IGFBP-3*)	TCA​TGC​CAA​GGA​CAG​CCA​GCG	CCT​CCA​TTT​CCC​GGC​GGC​AG	NM_001159276.1
Insulin receptor A (*IR A*)	CCCGAAGACCGACTCTCA	AGGCCTGGGGATGAAAAC	Y16093.1
Insulin receptor B (*IR B*)	CCG​AAG​ACC​GAC​TCT​CAG​AT	CAA​CAG​GGC​CTG​AAG​ATG​AT	Y16092.1
Beta-2-microglobulin	CTT​GGT​CCT​TCT​CGG​GCT​G	ATC​TTC​TGG​CGG​GTG​TCT​TG	NM_001009284.2
Hydroxymethylbilan synthase	AGC​CCA​GCT​GCA​GAG​AAA​G	CAG​CCG​TGT​GTT​GAG​GTT​TC	XM_042232961.1
Ribosomal protein L37a	ACC​AAG​AAG​GTC​GGA​ATC​GT	GGC​ACC​ACC​AGC​TAC​TGT​TT	XM_027965159
Ribosomal protein S15	ATC​ATT​CTG​CCC​GAG​ATG​GTG	CGG​GCC​GGC​CAT​GCT​TTA​CG	NM_001018.2

### Statistics

Data were analyzed using GraphPad Prism (San Diego, CA). One-way ANOVA was used to determine the effect of oIGF-1 on myoblast proliferation *in vitro*; Bonferroni’s multiple comparisons test was used for *post hoc* analysis. Paired t-tests were used to compare fetal body and organ weights, FSR, EdU positivity, and mRNA expression of IGF-1 targets between twin animals that received chronic (1 week) infusion (oIGF-1, SAL); Wilcoxon matched pairs signed rank test was used for non-parametric data. Two-way ANOVA with repeated measures or a mixed-effects model was used to determine the effect of time (infusion day), group (oIGF-1, SAL), and their interaction for repeated fetal blood measurements, including fetal blood gas, glucose, lactate, hormone, and amino acid concentrations. Bonferroni’s multiple comparisons test was used for *post hoc* analysis. Values are reported as mean ± SEM. A *p*-value of ≤0.05 was considered statistically significant.

## Results

### Myoblast proliferation *in vitro*

**FIGURE 2:**
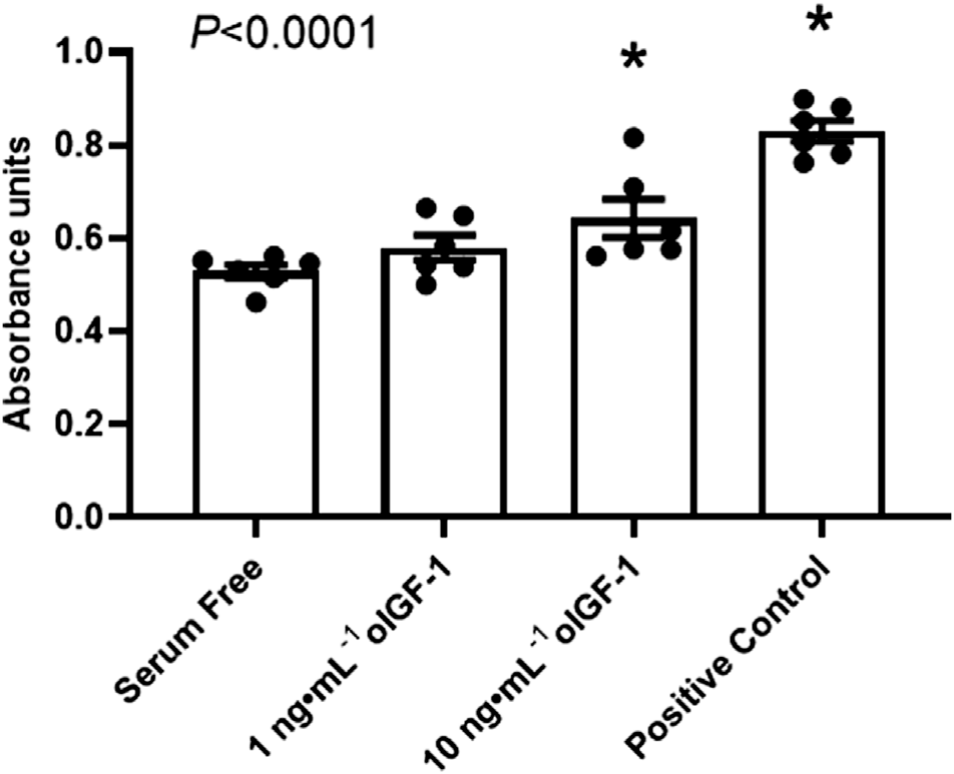
Myoblast proliferation *in vitro*. Myoblasts harvested from biceps femoris muscle from normally growing late gestation fetal sheep (n = 6) were exposed to serum free DMEM or 1 or 10 ng·mL^−1^ of recombinant ovine IGF-1 (oIGF-1) in DMEM for 72 h. Myoblasts were also exposed to DMEM with 10% FBS as a positive control. Myoblast proliferation was measured using 3-(4,5-dimethylthiazol-2-yl)-2,5-diphenyl tetrazolium bromide (MTT) assay. Significant effect by one-way ANOVA is indicated. *Indicates significantly different (*p* ≤ 0.05) from serum free DMEM by Bonferroni’s *post hoc* test.

As a positive control, FBS increased myoblast proliferation by 57% compared to DMEM alone. oIGF-1 at a concentration of 10 ng mL^−1^ in DMEM increased myoblast proliferation by 22% compared to DMEM alone (*p* = 0.045 by *post hoc* analysis) (Figure 2).

### Fetal oIGF-1 and insulin concentrations after acute (intraoperative) infusion of oIGF-1

**FIGURE 3:**
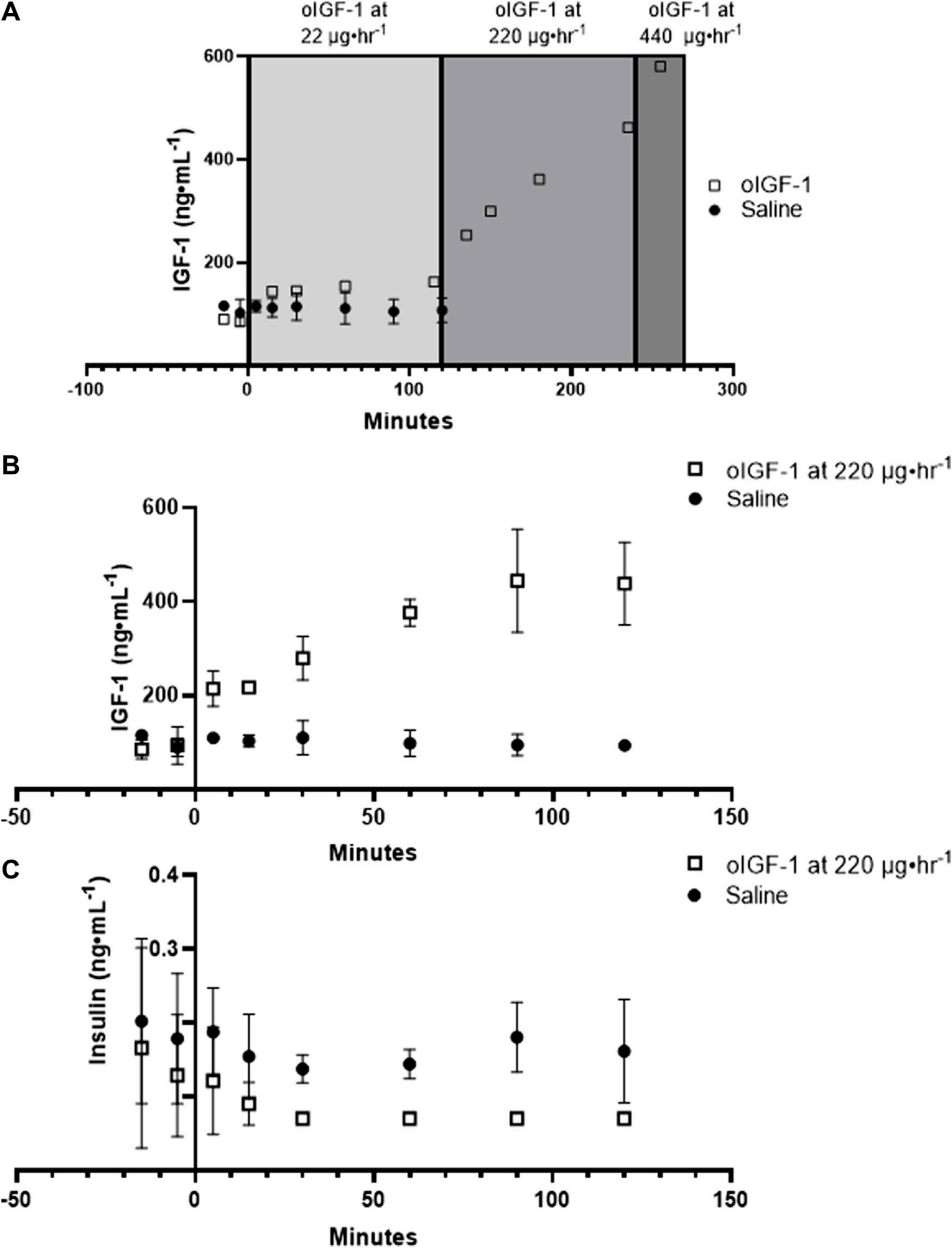
Fetal concentrations of recombinant ovine IGF-1 (oIGF-1) and insulin. **(A)** Dose response curve showing fetal plasma IGF-1 concentrations after infusion of oIGF-1 into a singleton fetus (open squares, n = 1) at 22, 220, and 440 μg·h^−1^. Fetal plasma IGF-1 concentrations are also shown after fetal infusion of saline (closed circles, n = 3) for reference. **(B)** Fetal plasma IGF-1 and **(C)** insulin concentrations after fetal infusion of either oIGF-1 at 220 μg·h^−1^ (open squares, n = 2) or saline (closed circles n = 2) into twin pairs.

oIGF-1 was infused into a singleton fetus at increasing concentrations to measure the dose response (Figure 3A). At 22 μg h^−1^, fetal concentrations of IGF-1 were similar to saline infused fetuses. Fetal concentrations of IGF-1 increased with infusion rates of 220 and 440 μg h^−1^, and at the end of the study, they were 5-fold higher than baseline. Next, in twin fetuses, oIGF-1 was infused at 220 μg h^−1^ for 2 hours in one twin fetus, and the other twin received a saline infusion. At the end of the infusion period, fetal IGF-1 concentrations were 3.5-fold higher in oIGF-1 infused fetuses compared to saline infused fetuses (Figure 3B). Because IGF-1 infusion decreases fetal insulin concentrations (Liechty et al., 1996; Lok et al., 1996; Boyle et al., 1998; White et al., 2018; Stremming et al., 2021; White et al., 2021), plasma insulin concentrations were obtained. At the end of the infusion period, fetal plasma insulin concentrations were 89% lower in oIGF-1 infused fetuses compared to fetuses that received a saline infusion (Figure 3C).

### Anabolic signaling pathways after acute infusion of oIGF-1

**FIGURE 4:**
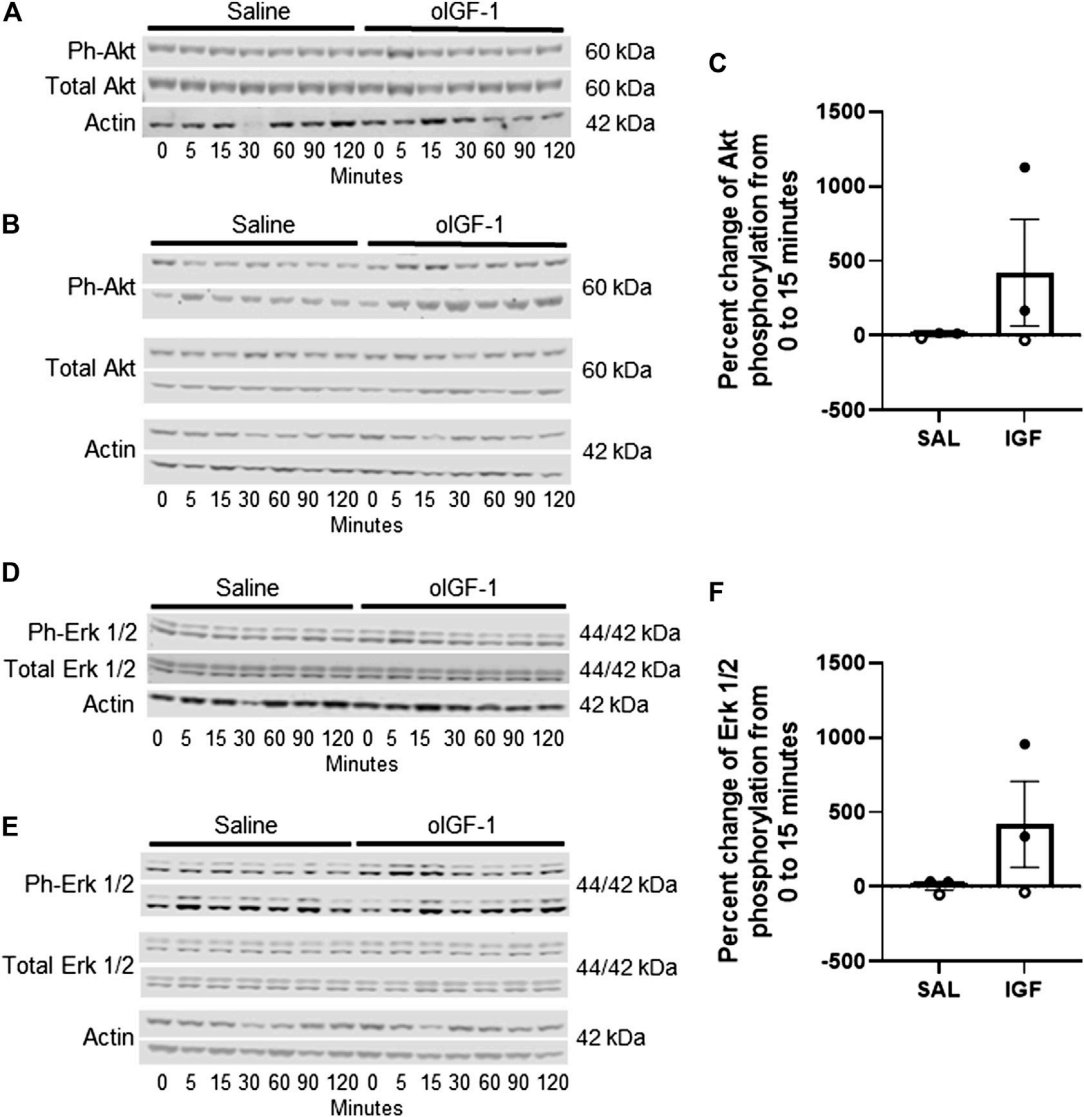
Protein expression of anabolic targets in biceps femoris muscle after acute infusion of recombinant ovine IGF-1 (oIGF-1). Blots of phosphorylated (Ser473) and total Akt in **(A)** 1 pair of twin animals that received oIGF-1 at 22 μg·h^−1^ or saline and **(B)** 2 pairs of twin animals that received oIGF-1 at 220 μg·h^−1^ or saline. **(C)** Graph represents the percent change in phosphorylation of Akt from 0 to 15 min of infusion. Open circles represent the twin pair that received oIGF-1 at 22 μg h^−1^ or saline and closed circles represent the twin pairs that received oIGF-1 at 220 μg·h^−1^ or saline. Blots of phosphorylated (Thr202/Tyr204) and total Erk 1/2 in **(D)** 1 pair of twin animals that received oIGF-1 at 22 μg·h^−1^ or saline and **(E)** 2 pairs of twin animals that received oIGF-1 at 220 μg·h^−1^ or saline. **(F)** Graph represents the percent change in phosphorylation of Erk 1/2 from 0 to 15 min of infusion. Open circles represent the twin pair that received oIGF-1 at 22 μg·h^−1^ or saline and closed circles represent the twin pairs that received oIGF-1 at 220 μg·h^−1^ or saline.

Phosphorylation of Akt (Ser 473) and Erk 1/2 (Thr 202/Tyr 204) (Figure 4) increased by 40-fold and 16-fold, respectively, in skeletal muscle from 0 to 15 min of infusion of oIGF-1 at 220 μg h^−1^ compared to animals that received a saline infusion. However, due to a small number of animals (n = 2 per group), this was not statistically significant.

### Fetal characteristics of sheep after chronic (1 week) infusion of oIGF-1

Fetal body weight was similar between oIGF-1 and SAL (Table 2) as was crown rump length. Fetal heart, kidney, adrenal gland, and spleen weights were significantly higher in oIGF-1 (*p* < 0.05); when organ weights were normalized to body weight, these organ weights remained significantly higher in oIGF-1 (data not shown). Weight of the brain, lungs, and liver were similar between groups, even when normalized to fetal weight. Weights of the individual skeletal muscles, including gastrocnemius, tibialis anterior, and flexor digitorum superficialis, were also similar between groups. When normalized to fetal weight, tibialis anterior was 20% lighter in oIGF-1 (oIGF-1: 0.81 ± 0.08 g kg^−1^ vs. SAL: 1.01 ± 0.03 g kg^−1^, *p* < 0.05).

**TABLE 2:** Fetal body and organ weights after 1-week infusion of oIGF-1 or saline (SAL).

	SAL (n = 6)	oIGF-1 (n = 6)	*p*-value
% Male	50%	33%	
Fetal weight (kg)	3.021 ± 0.198	3.032 ± 0.138	0.9090
Crown rump length (cm)	49.2 ± 1.3	48.3 ± 1.3	0.2543
Hindlimb length (cm)	38.2 ± 1.0	37.4 ± 1.6	0.6404
Brain (g)	52.0 ± 1.5	52.0 ± 1.1	0.9728
Heart (g)	21.9 ± 1.7	27.6 ± 1.9	0.0202
Liver (g)	63.4 ± 4.3	68.7 ± 6.8	0.4391
Lungs (g)	116.3 ± 9.2	119.4 ± 5.9	0.6612
Kidneys (g)	19.3 ± 1.5	23.9 ± 1.4	0.0072
Adrenal glands (g)	0.38 ± 0.03	0.57 ± 0.03	0.0011
Spleen (g)	5.8 ± 0.9	8.2 ± 0.7	0.0146
Gastrocnemius (g)	7.1 ± 0.4	7.4 ± 0.3	0.5093
Flexor digitorum superficialis (g)	2.5 ± 0.3	2.0 ± 0.4	0.8438
Tibialis anterior (g)	3.1 ± 0.2	2.5 ± 0.3	0.1128

Values represent mean ± SEM. *p*-values determined by paired *t*-test or wilcoxon matched pairs signed rank test.

**FIGURE 5:**
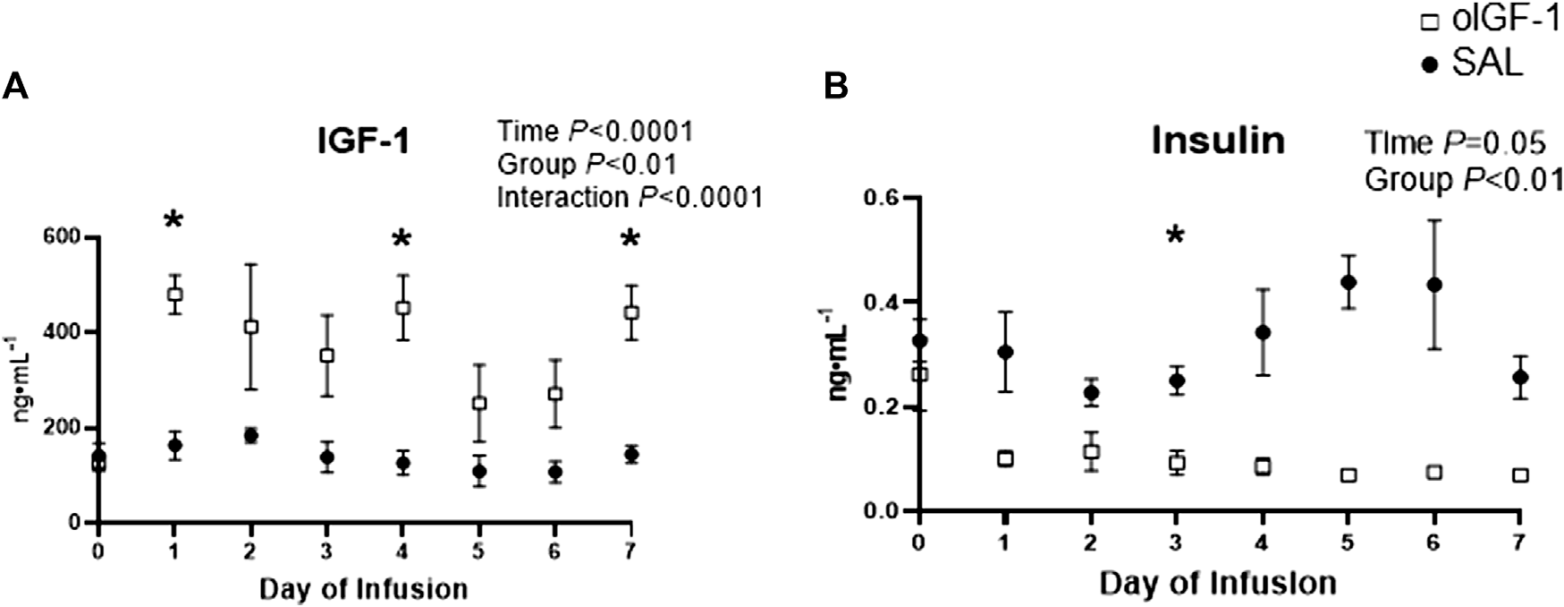
Fetal concentrations of IGF-1 and insulin during chronic recombinant ovine IGF-1 (oIGF-1) infusion. Fetal plasma concentrations of **(A)** IGF-1 and **(B)** insulin in fetal sheep that received either oIGF-1 (n = 6, open squares) or saline (SAL, n = 6, closed circles) infusion for 1 week. Significant effects by mixed model ANOVA [time, group (oIGF-1, SAL), and interaction] are indicated. *Indicates significantly different (*p* ≤ 0.05) between oIGF-1 and SAL by Bonferroni’s *post hoc* test.

Fetal IGF-1 concentrations increased in animals that received oIGF-1 infusion for 1 week (Figure 5A); at the end of the infusion period, fetal IGF-1 concentrations in oIGF-1 were 204% higher than in SAL (*p* < 0.01 for group effect and *p* < 0.05 for *post hoc* comparison of final concentrations). Fetal insulin concentrations decreased during oIGF-1 infusion compared to SAL (Figure 5B), and by the end of the infusion, they were 88% lower in oIGF-1 than SAL (*p* < 0.01 for group effect and *p* = 0.08 for *post hoc* comparison of final concentrations). However, fetal glucose concentrations were similar between groups (Table 3). Fetal lactate, cortisol, and norepinephrine concentrations, and blood gas measures were similar between groups (Table 3). Fetal concentrations of some amino acids, including arginine, histidine, lysine, and ornithine were lower in oIGF-1 compared to SAL at the end of the study (Table 4, *p* < 0.05 for interaction of group and time and *post hoc* comparison of final concentrations). At the end of the infusion period, fetal concentrations of summed amino acids were lower in oIGF-1 (oIGF-1: 2,930 ± 195 μmol L^−1^ vs SAL: 3,495 ± 223 μmol L^−1^, *p* = 0.05).

**TABLE 3:** Fetal glucose, lactate, blood gas, and hormone concentrations after 1-week infusion of oIGF-1 or saline (SAL).

	SAL (n = 6)		oIGF-1 (n = 6)		*p*-values		
	Baseline	Final	Baseline	Final	Time	Group	Interaction
Glucose (mg·dL^−1^)	15.63 ± 0.53	14.36 ± 0.71	16.53 ± 1.11	13.34 ± 1.82	**0.0406**	0.9648	0.3365
Lactate (mmol·L^−1^)	1.54 ± 0.06	1.42 ± 0.12	1.58 ± 0.03	1.68 ± 0.26	0.9303	0.3062	0.4887
pH	7.34 ± 0.01	7.32 ± 0.02	7.34 ± 0.01	7.33 ± 0.02	0.2247	0.9158	0.5541
P_a_CO_2_ (mmHg)	48.78 ± 1.11	51.40 ± 1.68	49.40 ± 0.70	51.85 ± 1.11	**0.0427**	0.6899	0.9407
P_a_O_2_ (mmHg)	21.18 ± 0.85	20.15 ± 1.27	21.02 ± 1.41	19.85 ± 1.19	0.3310	0.8616	0.9518
Hematocrit (%)	34.3 ± 1.19	33.25 ± 0.90	34.25 ± 0.73	32.55 ± 0.90	**0.0180**	0.7842	0.4807
O_2_ saturation (%)	52.32 ± 2.69	48.57 ± 5.26	49.53 ± 5.19	47.47 ± 5.65	0.4683	0.7386	0.8317
O_2_ content (mmol·L^−1^)	3.57 ± 0.21	3.22 ± 0.37	3.35 ± 0.35	2.93 ± 0.32	0.1385	0.5293	0.8914
Cortisol (ng·mL^−1^)Δ	24.8 ± 7.2	19.7 ± 4.2	22.5 ± 6.1	25.6 ± 5.9	0.8731	0.7704	0.5184
Norepinephrine (pg·mL^−1^)	1,022.5 ± 353.1	897.2 ± 394.6	1,144.2 ± 400.0	1,455.0 ± 301.9	0.4295	0.5148	0.0816

Values represent mean ± SEM. *p*-values determined by two-way repeated measures ANOVA for the effects of time, group (SAL, oIGF-1), and interaction. Δ indicates n = 4 per group. Bolded values indicates significant *p* values.

**TABLE 4:** Fetal concentrations of amino acids after 1-week infusion of oIGF-1 or saline (SAL).

	SAL (n = 6)		oIGF-1 (n = 6)		*p*-values		
	Baseline	Final	Baseline	Final	Time	Group	Interaction
Alanine	211 ± 13	246 ± 22	192 ± 15	196 ± 46	0.4039	0.2963	0.4935
Arginine	90 ± 12	122 ± 5	87 ± 14	68 ± 9*	0.3360	0.0563	**0.0035**
Asparagine	33 ± 3	43 ± 6	32 ± 2	36 ± 5	0.0559	0.4549	0.3572
Aspartate	28 ± 7	27 ± 5	25 ± 6	21 ± 2	0.3827	0.5244	0.5745
Cysteine	18 ± 2	20 ± 2	16 ± 2	15 ± 2	0.7171	0.1941	0.4644
Glutamate	28 ± 3	29 ± 4	24 ± 2	24 ± 3	0.9467	0.2966	0.7835
Glutamine	390 ± 19	416 ± 43	361 ± 14	304 ± 36	0.4627	0.0967	0.0664
Glycine	318 ± 54	378 ± 57	325 ± 43	460 ± 60	0.0521	0.4920	0.4207
Histidine	37 ± 5	51 ± 7	32 ± 4	32 ± 5*	**0.0285**	0.1122	**0.0285**
Isoleucine	84 ± 6	88 ± 5	82 ± 8	76 ± 6	0.8406	0.4007	0.2311
Leucine	110 ± 8	124 ± 8	105 ± 9	101 ± 9	0.4103	0.1852	0.1767
Lysine	60 ± 10	85 ± 12	55 ± 6	44 ± 7*	0.1691	0.0938	**0.0037**
Methionine	93 ± 9	86 ± 13	89 ± 10	68 ± 9	**0.0331**	0.4270	0.2791
Ornithine	53 ± 6	65 ± 7	54 ± 8	38 ± 4*	0.6517	0.1014	**0.0244**
Phenylalanine	97 ± 5	110 ± 7	97 ± 4	109 ± 9	**0.0187**	0.9547	0.9979
Proline	116 ± 7	151 ± 17	119 ± 8	110 ± 12	0.2826	0.1313	0.0873
Serine	531 ± 41	530 ± 38	521 ± 43	433 ± 41	0.2314	0.2656	0.2435
Taurine	78 ± 19	97 ± 42	72 ± 14	77 ± 10	0.3791	0.6822	0.5940
Threonine	274 ± 43	293 ± 40	234 ± 27	217 ± 26	0.9546	0.2471	0.2152
Tryptophan	46 ± 2	49 ± 2	40 ± 4	43 ± 2	0.2085	0.1196	0.8083
Tyrosine	120 ± 8	120 ± 12	126 ± 5	117 ± 9	0.4822	0.8971	0.4885
Valine	329 ± 18	365 ± 24	320 ± 24	313 ± 21	0.5671	0.1516	0.3846
Summed	3,147 ± 148	3,495 ± 223	3,009 ± 115	2,903 ± 195	0.3657	0.1164	0.0626

Amino acid concentrations are reported as µmol·L^−1^. Values represent mean ± SEM. p-values determined by two-way repeated measures ANOVA for the effects of time, group (SAL, oIGF-1), and interaction. *Indicates significantly lower (p ≤ 0.05) compared to final SAL by Bonferroni’s *post hoc* test. Bolded values indicates significant *p* values.

### *In vivo* muscle growth and signaling after chronic infusion of oIGF-1

**FIGURE 6:**
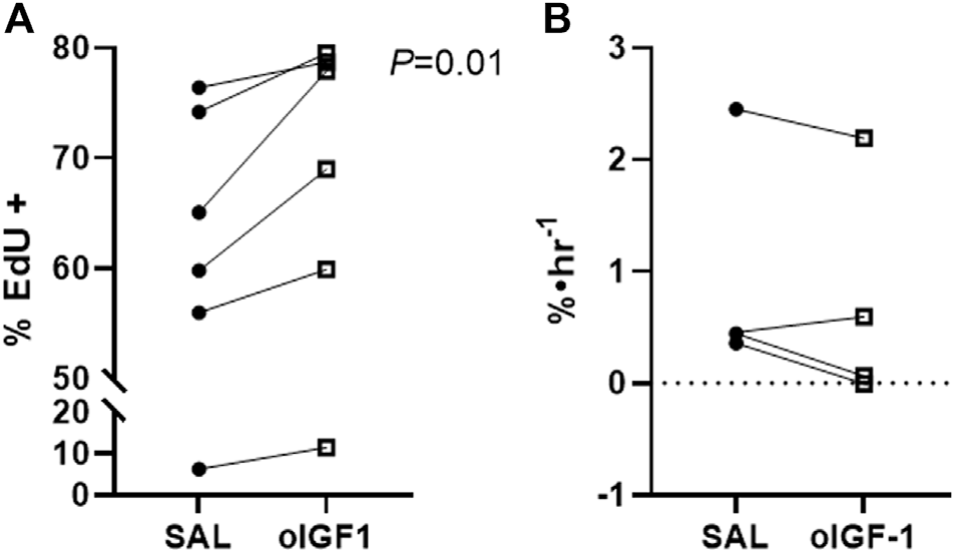
Myoblast proliferation and fractional synthetic rate (FSR) after chronic infusion of recombinant ovine IGF-1 (oIGF-1). **(A)** Percent of myoblasts in biceps femoris (BF) muscle that were positive for EdU by flow cytometry in fetal sheep treated with oIGF-1 (n = 6) or saline (SAL, n = 6). Twin pairs are connected. *p*-value indicated was determined by paired *t*-test. **(B)** FSR in BF from fetal sheep treated with oIGF-1 (n = 4) or SAL (n = 4). Twin pairs are connected.

The percentage of EdU positive myoblasts was 11% higher in oIGF-1 compared to SAL (*p* = 0.01), which signals increased myoblast proliferation (Figure 6A). The fractional synthetic rate was similar in oIGF-1 (n = 4) compared to SAL (n = 4) after a 1-week infusion of oIGF-1 (Figure 6B). Expression of both total and phosphorylated Akt (Ser 473) and total and phosphorylated Erk 1/2 (Thr202/Tyr204) was similar in SAL and oIGF-1 (data not shown).

### Transcriptional response to chronic infusion of oIGF-1 in skeletal muscle and liver

**FIGURE 7:**
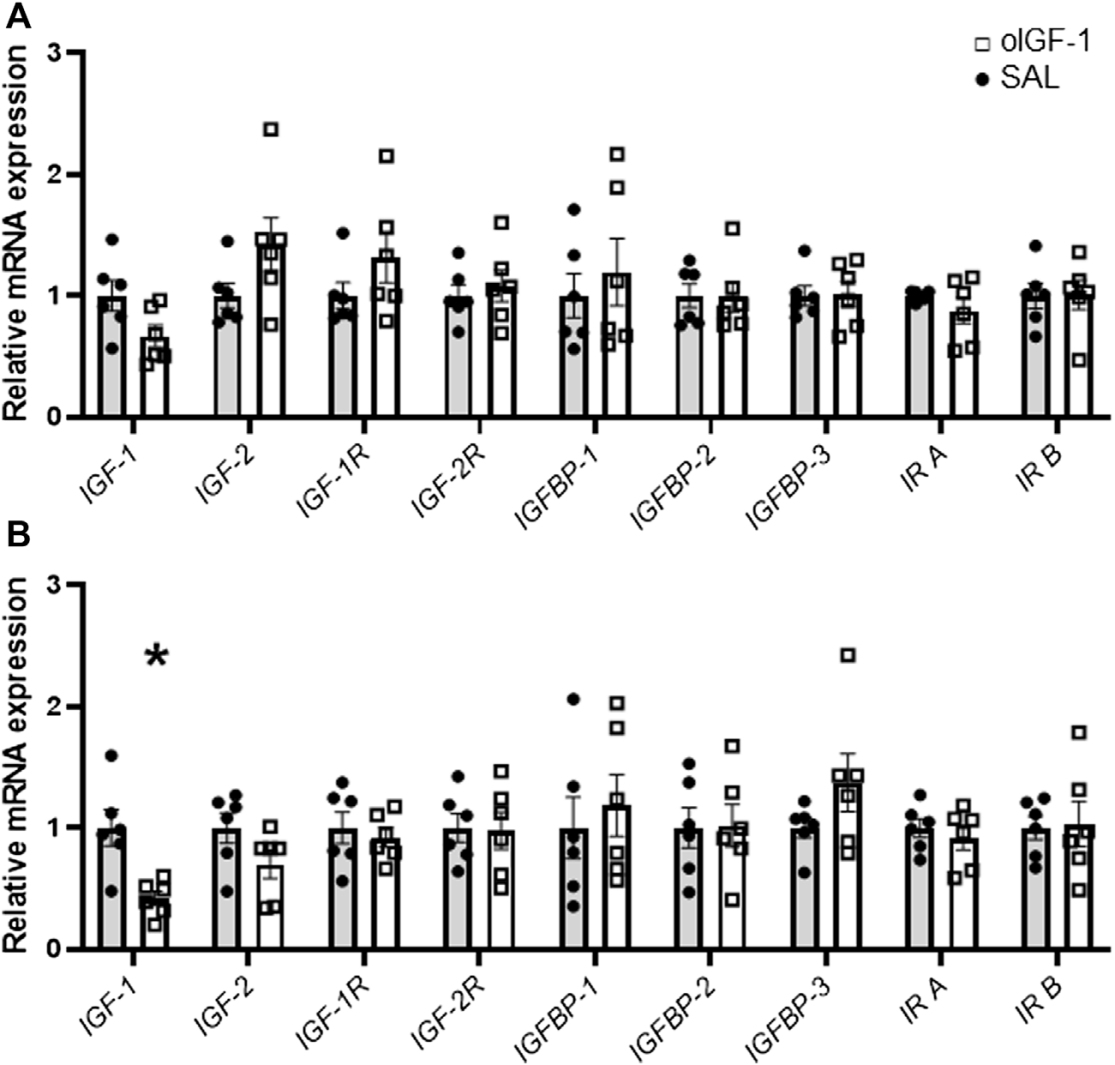
mRNA expression of IGF related targets in biceps femoris muscle and liver after chronic recombinant ovine IGF-1 (oIGF-1) infusion. mRNA expression of IGF related targets in **(A)** muscle and **(B)** liver from fetal sheep that received a 1-week infusion of either oIGF-1 (n = 6, open squares) or saline (SAL, n = 6, closed circles). Individual data points and means ± SEM are shown. *Indicates significantly different (*p* ≤ 0.05) between SAL and oIGF-1 as determined by paired *t*-test.

Liver mRNA expression of *IGF-1* was 58% lower in oIGF-1 compared to SAL infused fetuses (*p* = 0.01) (Figure 7). *IGF-2* expression tended to be lower in oIGF-1 (*p* = 0.07). Expression of *IGF-1* and *IGF-2* in skeletal muscle was similar between groups. Expression of *IGF-1 receptor*, *IGF-2 receptor,* and *insulin receptor A* and *B* was similar in SAL and oIGF-1 in muscle and liver. Expression of *IGFBP-1*, *IGFBP-2*, and *IGFBP-3* was also similar in SAL and oIGF-1 in both muscle and liver.

## Discussion

In this study, we demonstrated that oIGF-1, like LR3 IGF-1 (Stremming et al., 2021), increased *in vitro* myoblast proliferation, *in vivo* myoblast proliferation, and selective fetal organ weight, but it did not increase fetal body weight. Similar to infusion of LR3 IGF-1, fetal oIGF-1 infusion for 1 week also attenuated fetal insulin concentrations, which has been previously reported (Liechty et al., 1996; Lok et al., 1996; Boyle et al., 1998; White et al., 2018; Stremming et al., 2021; White et al., 2021); however, fetal glucose concentrations in oIGF-1 are maintained similar to control animals. Fetal plasma amino acid concentrations were also lower after infusion of oIGF-1, which is consistent with infusion of LR3 IGF-1 (Stremming et al., 2021). Interestingly, mRNA expression of *IGFBPs 1–3* in the liver, a primary site of production for IGFBPs (Allard and Duan, 2018; Bach, 2018), and in skeletal muscle, was similar in SAL and oIGF-1. In addition, fetal oIGF-1 plasma concentrations were measurable with ELISA, which could not be done with LR3 IGF-1; this will be valuable for understanding associations between the magnitude of fetal IGF-1 concentrations and growth. Together, these characteristics make oIGF-1 an invaluable tool to test the growth effects of IGF-1 using a sheep model.

In most mammals, the *IGF-1* gene contains 6 exons and 5 introns (Rotwein, 2017). In a study examining the DNA sequence of *IGF-1* in 25 mammals, the DNA sequence in exons one to four was generally similar among many mammals. However, the sequences of exons 5 and 6 varied among mammals. The *IGF1* gene also encodes promoter sites, signal peptides, and C terminal extension peptides. Although the protein sequence of mature IGF-1 in sheep is almost identical to human, it does differ by one amino acid: a proline in the human sequence is replaced by an alanine in the sheep sequence (Rotwein, 2017). Because proline contains a unique cyclic structure, it may cause a bend in the protein. Thus, replacing a proline with an alanine could potentially cause structural changes in the peptide. In addition to the differences between native human and sheep IGF-1, the peptide of human recombinant LR3 IGF-1, an IGF-1 analog frequently used in research studies, differs from native human and sheep IGF-1; LR3 IGF-1 contains an additional 13 amino acids on the N terminal, and it contains an amino acid substitution of the third amino acid from glutamic acid, which is positively charged, in the native human and sheep sequences to arginine, which is negatively charged, in LR3 IGF-1. Thus, due to these differences between native sheep IGF-1 and human recombinant LR3 IGF-1, we developed a sheep specific IGF-1 to be infused into fetal sheep to test its effect on fetal growth.

To test the efficacy of oIGF-1 in stimulating organ-specific growth in fetal sheep, we first exposed cultured fetal myoblasts to oIGF-1 for 72 h and then tested *in vivo* signaling effects in skeletal muscle. IGF-1 is known to stimulate both proliferation and differentiation of myoblasts (Singleton and Feldman, 2001). In the fetus, IGF-1 receptor knockout mice had fewer myocytes (Liu et al., 1993); postnatally, IGF-1 receptor knockout mice had smaller myofibers and fewer myonuclei compared to wild type animals (Mavalli et al., 2010). Indeed, oIGF-1 promoted *in vitro* myoblast proliferation in this study. Downstream of the IGF-1 receptor are two distinct signaling pathways, phosphatidylinositol-3-kinase (PI3K) and mitogen-activated protein kinase (MAPK). Inhibition of targets in both the MAPK and PI3K pathways inhibit myoblast proliferation and differentiation in L6A1 myoblasts and myoblasts cultured from chicken embryos (Coolican et al., 1997; Yu et al., 2015). Interestingly, we showed that in acute BF muscle biopsies, phosphorylation of Erk 1/2 and Akt, downstream components of the MAPK and PI3K pathways, respectively, was increased within 15 minutes of oIGF-1 infusion into the sheep fetus. However, phosphorylation of both proteins returned to baseline by the end of the infusion period (120 min). This may reflect a brief activation of these signaling pathways in response to oIGF-1. After infusion of oIGF-1 to the sheep fetus for 1 week, there was no difference in phosphorylation of Akt or Erk 1/2; this may reflect habituation of the fetus to the continuous infusion of oIGF-1. Despite a lack of differences in signaling pathways between animals that received a 1-week infusion of either oIGF-1 or saline, oIGF-1 animals did have increased *in vivo* myoblast proliferation in BF compared to SAL animals. However, skeletal muscle weights of hindlimb muscles including gastrocnemius, tibialis anterior, and flexor digitorum superficialis were similar between groups, which may be related to the lack of an increase in the muscle fractional synthetic rates in response to oIGF-1. This is consistent with our previous studies using LR3 IGF-1 (Stremming et al., 2021).

LR3 IGF-1 infusion has been shown to increase the weights of several fetal organs, including heart, spleen, and adrenal glands without increasing the weight of the whole fetus (Sundgren et al., 2003; Stremming et al., 2021). We have now shown that oIGF-1 also increases the weight of fetal organs, including heart, spleen, adrenal glands, and kidneys without increasing fetal body weight. Despite a selective increase in fetal organ size, IGF-1 receptor is known to be present in many fetal organs in cows, including the brain, heart, liver, kidney, lung muscle, and testis (Ghanipoor-Samami et al., 2018). Previous studies have shown that IGF-1 mediated fetal heart growth is stimulated by increased proliferation of cardiomyocytes as opposed to hypertrophy (Sundgren et al., 2003), whereas adrenal gland growth may be due to increased cellular hypertrophy (Ross et al., 2007). Whether enhanced spleen and kidney growth in response to oIGF-1 is caused by increased cellular proliferation or hypertrophy requires further study. Infusion of either LR3 IGF-1 or oIGF-1 did not increase the weight of the fetal liver or lungs. The liver is a major source of IGF-1 production (Gluckman and Pinal, 2003; Setia et al., 2006; Hellström et al., 2016), however, protein expression of IGF-1 receptor decreases as hepatocytes develop, and mature hepatocytes do not express IGF-1 receptor (Waraky et al., 2016). Thus IGF-1 infusion may not stimulate liver growth. It is unclear why oIGF-1 infusion does not stimulate lung growth as IGF-1 is critical for lung development; mice with knockouts of IGF-1 receptor have lung hypoplasia and delayed lung maturation (Epaud et al., 2012). We also showed that there was no difference in brain size between oIGF-1 and SAL, even when normalized to fetal body weight. Previously, we showed that normalized brain weight was lower in LR3 IGF-1 animals compared to control (Stremming et al., 2021). Thus, it is reassuring that oIGF-1 infusion does not limit brain growth.

It is unclear why IGF-1 infusion, including LR3 IGF-1 or oIGF-1, to fetal sheep does not consistently increase fetal body weight despite increased fetal organ weight. One possibility may involve concurrent downregulation of insulin concentrations and potential synergy with amino acid supply and metabolism. LR3 IGF-1 infusion decreases fetal insulin and amino acid concentrations (Liechty et al., 1996; Lok et al., 1996; Boyle et al., 1998; White et al., 2018; Stremming et al., 2021; White et al., 2021), which may limit fetal growth potential. Consistent with previous studies, we also demonstrated that fetal insulin concentrations were reduced after infusion of oIGF-1 for 1 week. Although the exact mechanism to explain reduced insulin concentrations after IGF-1 infusion is not well understood, our lab has previously shown that infusion of LR3 IGF-1 attenuates glucose stimulated insulin secretion *in vivo* and islet cell secretion of insulin *in vitro* (White et al., 2021). Pancreatic insulin content is also increased, suggesting an inhibition of insulin secretion rather than production (White et al., 2018). Fetal arterial glucose, lactate, and oxygen concentrations were unchanged in oIGF-1 infused fetuses after a 1-week infusion. However, fetal concentrations of amino acids were lower after infusion of oIGF-1 for 1 week, which is consistent with our previous study using LR3 IGF-1 (Stremming et al., 2021). The identities of these amino acids differed between studies. Interestingly, in this study, fetal arginine, histidine, lysine, and ornithine were lower in fetal oIGF-1 compared to SAL. Arginine, lysine, and ornithine are known to stimulate growth hormone secretion; some studies have shown that histidine also has this effect (Chromiak and Antonio, 2002). Postnatally, growth hormone is a critical stimulator of IGF-1 secretion. However, this is not thought to be true in the fetus despite detectable concentrations of growth hormone in the fetal circulation (Fowden, 1995; Randhawa and Cohen, 2005; Setia et al., 2006). Thus, it is unclear if lower fetal concentrations of these amino acids are connected to growth hormone concentrations in the setting of significantly elevated fetal IGF-1 concentrations. Studies are currently ongoing to understand the relationship between IGF-1, insulin, and amino acid concentrations and how these hormones and nutrients coordinate to regulate fetal growth.

One of the limitations of using LR3 IGF-1 is that it is not easily measured in the fetal circulation. In our previous studies using LR3 IGF-1, we were able to measure endogenous fetal IGF-1 concentrations by ELISA but not fetal concentrations of the LR3 IGF-1 infusate. Endogenous concentrations of IGF-1 were 45% lower in animals that received infusion of LR3 IGF-1 compared to control, likely due to suppression of endogenous IGF-1 secretion in response to exogenous LR3 IGF-1 infusion (Stremming et al., 2021; White et al., 2021). In this study, we showed that fetal IGF-1 concentrations rose within minutes of infusion of oIGF-1 at 220 μg h^−1^. After 1 week of oIGF-1 infusion, fetal IGF-1 concentrations were 204% higher than SAL animals. Knowledge of actual fetal IGF-1 concentrations after IGF-1 infusion is beneficial in two ways. First, this will allow for the development of specific dosing regimens of fetal IGF-1 for therapeutic purposes. Second, it allows for evaluation of the correlations of IGF-1 with other hormones, including insulin, and nutrients in order to understand the relationships between these substances.

One of the well-known characteristics of LR3 IGF-1 is its limited capacity for binding IGFBPs, thus making it more available for receptor binding. Previous studies have shown that infusion of recombinant human IGF-1 to the sheep fetus for 3 hours yielded small (≤6%) but statistically significant increases in fetal plasma concentrations of IGFBP-1, IGFBP-2, and IGFBP-3 despite large (≥74%) increases in IGF-1 concentrations (Lee et al., 1997). After an 8-h infusion of recombinant human IGF-1 to the sheep fetus, IGFBP-1 and IGFBP-3 plasma concentrations were increased by at least 30% (Shen et al., 2001). Insulin, amino acid, and glucose concentrations were experimentally maintained at baseline values in this study. In the fetal liver, mRNA expression of *IGFBP-1* also increased with infusion of recombinant human IGF-1 in the setting of normal fetal insulin and nutrient concentrations, but expression of *IGFBP-3* did not change (Shen et al., 2001). While we did not measure fetal concentrations of circulating IGFBPs, we did measure mRNA expression of *IGFBPs 1–3* in the liver and skeletal muscle. The liver is thought to be the primary source of IGFBP production, although the IGFBP genes are expressed throughout the body (Allard and Duan, 2018; Bach, 2018; Ghanipoor-Samami et al., 2018). We showed that there was no difference in mRNA expression of *IGFBP-1, IGFBP-2,* or *IGFBP-3* in the liver or skeletal muscle after a 1-week infusion of oIGF-1 compared to SAL in the setting of fetal hypoinsulinemia and lower amino acid concentrations. Factors such as chronic nutrient deprivation, hypoxia, insulin concentrations, growth hormone concentrations, and glucocorticoid concentrations are known to regulate IGFBP-1 and IGFBP-3 expression (Suwanichkul et al., 1994; Estívariz and Ziegler, 1997; Phillips et al., 1998; Maures and Duan, 2002; Allard and Duan, 2018); a combination of these factors, such as lower fetal insulin and amino acid concentrations may be important in our current study. Phosphorylation of IGFBPs has shown to greatly impact the binding capability of IGFBPs (Gupta, 2015), and factors such as amino acid (leucine) deprivation may regulate IGFBP phosphorylation (Seferovic et al., 2009). Post-translational modifications could impact the function of IGFBPs in the setting of preserved mRNA expression. More research is needed to understand the impact of IGF-1 infusion on fetal IGFBP availability and posttranslational modifications that may affect activity.

### Perspectives and significance

IGF-1 is being evaluated as a potential therapeutic option to mitigate abnormal fetal growth in human pregnancies. However, prior to utilizing IGF-1 in the human fetus, more work is necessary to understand how IGF-1 facilitates fetal growth and fetal organ growth, how IGF-1 coordinates fetal nutrient availability, and how IGF-1 interacts with other critical growth hormones, including insulin. To advance our knowledge of how IGF-1 promotes fetal growth, it is important to mimic physiologic conditions whenever possible. The sheep model of IUGR is a powerful animal model to study not only the fundamental physiology of IUGR but also to test potential treatment options to restore growth trajectories. Exogenous native sheep IGF-1 will not only undergo physiologic binding and regulation in the sheep fetus but will also be measurable in the lab to allow for better fundamental understanding of how IGF-1 supports fetal growth. Using this novel development in sheep to fill this critical knowledge gap may ultimately lead to developments to improve fetal growth in humans.

## Data Availability

The original contributions presented in the study are included in the article/supplementary material, further inquiries can be directed to the corresponding author.
